# A Reply

**Published:** 1875-12

**Authors:** 


### EDITORIAL. ETC.

---

*A Reply.*—In the September number of this JOURNAL we published an article from the *New York Express*, concerning the late meeting of the American Dental Convention, at Long Branch, without knowing to whom this article referred, and with no intention whatever of injuring the aggrieved party or the Society of which he is an officer.

The only reason we had for noticing the article in question, was to give an opportunity for a reply, two of which we now publish. What was said by us concerning the American Dental Convention, apart from the article which appeared in the New York paper, was nothing more than a statement concerning its present condition, which we believed to be a true one, and we are pleasedto learn thatin some matters the opinionwe had formed and the knowledge gained from others, [never having attended a meeting since the formation of the American Dental Association,] was incorrect, and that it is in a more prosperous condition than we supposed was the case. Personally we have the same good feeling for this Society, as for any other striving to advance the status of our profession. The following replies are from gentlemen well versed in all that relates to the working of the American Dental Convention :

" In the September number of the AMERICAN JOURNAL OF DENTAL SCIENCE, under the head of Editorial, you have transcribed from the *New York Express* an anonymous communication, to which the officers of the Convention did not deem proper to reply. And in which the writer, after he '*eulogises* himself and others in a *self-praising* manner,' hints that if they had been the leaders, Capt. Leland, of the Ocean Hotel, would have been honored by over five hundred members of the dental profession. Now it is well known that the American Dentla Association, backed by the dental societies throughout the United States, together with the dental colleges, finds it difficult to muster even one hundred dentists.

The gentleman referred to as a defunct dentist, for years has had and still has a lucrative practice in New York city, and numbers among his patients eminent members of the Bar, the Clergy and Medical profession ; and it is not to be wondered at that he is an object of envy to such fellows as 'Wandering Willie." He was Chairman of the executive committee, and one of his duties was to arrange the business for the Convention, which he did to the satisfaction of nearly every member.

A few dentists who attended the Convention for the first time, attempted to introduce the matter referred to at an improper time, and not succeeding, they took offense.

The attendance was as large as usual, and very harmonious, and the proceedings are being published in the *Dental Cosmos.*

The Chairman in his report, stated that it had been customary to make an appropriation of ten dollars to the *Local Press*, as a slight compensation for reporting the proceedings ; with which large sum the *Local Press*, of Long Branch, was 'bought up.' "

The second reply is as follows, and refers to our statement concerning the present status of the Convention :

"You say the Convention has enemies in its midst ; its struggles for existence have been long and severe ; it has been patronized by a few New York dentists ; the association absorbed all its leading members," &c.

Allow me to say in all of these, you are very much mistaken ; the Convention has no enimies in its midst ; a few evil spirits came among us and tried to sow discord, but the seed took no root. The Convention has had no struggles for existence, but has gone on in the even tenor of its way; it is true that some years since, an effort was made to kill it, but the effort failed, and the Convention still lives. Members from the Convention from all parts of the Union, and across the water, have attended our meetings the last three years. Permit me to name a few who have been with us, in order to offset the statement " All the leading members, etc.

(A large number of names are here appended from the East, North, South and West, which the want of space compels us to omit.)

We regret that the matter became so personal, and that, without any knowledge of the writer, or the person referred to in the article in the *New York Express,* we were induced to publish the same, believing that by so doing, we should enable both sides to be heard without injustice to anyone.

